# Morita therapy for depression and anxiety (Morita Trial): study protocol for a pilot randomised controlled trial

**DOI:** 10.1186/s13063-016-1279-3

**Published:** 2016-03-24

**Authors:** Holly Victoria Rose Sugg, David A. Richards, Julia Frost

**Affiliations:** Complex Interventions Research Group, University of Exeter, University of Exeter Medical School, South Cloisters, St Luke’s Campus, Exeter, EX1 2 LU UK

**Keywords:** Morita therapy, Major depressive disorder, Mixed methods, Feasibility study

## Abstract

**Background:**

Morita Therapy, a psychological therapy for common mental health problems, is in sharp contrast to established western psychotherapeutic approaches in teaching that undesired symptoms are natural features of human emotion rather than something to control or eliminate. The approach is widely practiced in Japan, but untested and little known in the UK. A clinical trial of Morita Therapy is required to establish the effectiveness of Morita Therapy for a UK population. However, a number of methodological, procedural and clinical uncertainties associated with such a trial first require addressing.

**Methods/Design:**

The Morita Trial is a mixed methods study addressing the uncertainties associated with an evaluation of Morita Therapy compared with treatment as usual for depression and anxiety. We will undertake a pilot randomised controlled trial with embedded qualitative study. Sixty participants with major depressive disorder, with or without anxiety disorders, will be recruited predominantly from General Practice record searches and randomised to receive Morita Therapy plus treatment as usual or treatment as usual alone. Morita Therapy will be delivered by accredited psychological therapists. We will collect quantitative data on depressive symptoms, general anxiety, attitudes and quality of life at baseline and four month follow-up to inform future sample size calculations; and rates of recruitment, retention and treatment adherence to assess feasibility. We will undertake qualitative interviews in parallel with the trial, to explore people’s views of Morita Therapy. We will conduct separate and integrated analyses on the quantitative and qualitative data.

**Discussion:**

The outcomes of this study will prepare the ground for the design and conduct of a fully-powered evaluation of Morita Therapy plus treatment as usual versus treatment as usual alone, or inform a conclusion that such a trial is not feasible and/or appropriate. We will obtain a more comprehensive understanding of these issues than would be possible from either a quantitative or qualitative approach alone.

**Trial registration:**

Current Controlled Trials ISRCTN17544090 registered on 23 July 2015.

**Electronic supplementary material:**

The online version of this article (doi:10.1186/s13063-016-1279-3) contains supplementary material, which is available to authorized users.

## Background

Clinical depression and generalised anxiety disorder are the two most common mental health disorders [1], with one in six people in the UK experiencing such a disorder each year [2]. Together, depression and anxiety are estimated to cost the UK economy £17bn in lost output and direct health care costs annually, with a £9bn impact on the Exchequer through benefit payments and lost tax receipts [3].

**Table 1:** World Health Organization Trial Registration Data Set

Data category	Information
Primary registry and trial identifying number	Current Controlled Trials database
	ISRCTN17544090
Date of registration in primary registry	23-Jul-15
Secondary identifying numbers	N/A
Source(s) of monetary or material support	University of Exeter Medical School, UK
Primary sponsor	University of Exeter, UK
Secondary sponsor(s)	N/A
Contact for public queries	Holly Victoria Rose Sugg
	University of Exeter Medical School, UK
	h.v.s.sugg@exeter.ac.uk
Contact for scientific queries	Holly Victoria Rose Sugg
	University of Exeter Medical School, UK
	h.v.s.sugg@exeter.ac.uk
Public title	The Morita Trial
Scientific title	Morita Therapy for Depression and Anxiety: A Feasibility and Pilot Study
Countries of recruitment	UK
Health condition(s) or problem(s) studied	Depression
Intervention(s)	Morita Therapy
	Treatment as usual
Key inclusion and exclusion criteria	Ages eligible for study: ≥18 years;
	Sexes eligible for study: both;
	Accepts healthy volunteers: no
	Inclusion criteria: adult patient (≥18 years), current DSM Major Depressive Disorder
	Exclusion criteria: cognitive impairment, bipolar disorder or psychosis/psychotic symptoms, substance dependence, acute suicidal risk, current psychological therapy
Study type	Interventional
	Allocation: randomised intervention model
	Primary purpose: treatment
	Phase II
Date of first enrolment	Sep-15
Target sample size	72
Recruitment status	Recruiting
Primary outcome(s)	Depressive symptoms, generalised anxiety symptoms, quality of life, attitudes (at four-month follow-up); qualitative exploration of acceptability.
Key secondary outcomes	N/A

**Fig. 1:**
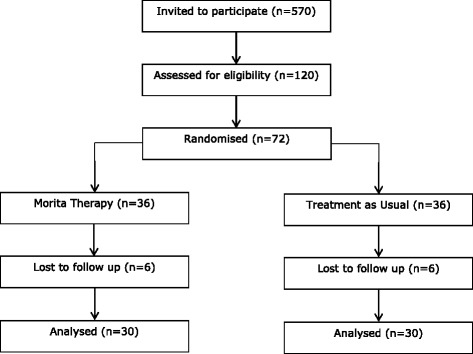
Consolidated Standards of Reporting Trials (CONSORT) diagram describing flow of participants through the study

**Fig. 2:**
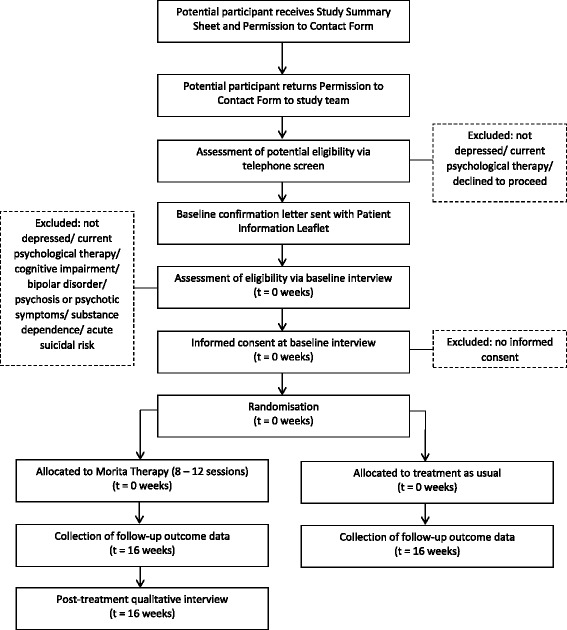
Schematic diagram describing timeline for participants in the study

Depression accounts for the greatest burden of disease among all mental health problems, and is the second-highest among all general health problems [4]. The lifetime prevalence of depression has been estimated at 16.2 %, and rates of co-morbidity and risk for suicide are high [5–7]. Depression is also recurrent, with over three quarters of people who recover from one episode experiencing at least one more [8].

Generalised Anxiety Disorder (GAD) affects between 2–5 % of the UK population at any one time, and accounts for up to 30 % of the mental health problems presented to General Practitioners (GPs) [2]. The lifetime prevalence of GAD has been estimated at 5.7 % [9]. Furthermore, the comorbidity between anxiety and depression make a strong contribution to the total disability attributed to mental disorders [1].

Medication and Cognitive Behavioural Therapy have the strongest evidence-base for treating these conditions, and are each recommended by the National Institute for Health and Care Excellence (NICE) [10, 11]. However, many patients are refractory to such interventions [12], with both depression and anxiety remaining chronic disorders despite treatment [1]. Recovery is only reached by 55–56 % of people receiving treatment through the large-scale UK initiative to provide NICE recommended psychological therapies (‘Improving Access to Psychological Therapies’ (IAPT)) [13, 14], thereby increasing the risk of future relapse and the maintenance of recurring and chronic problems [15].

Thus, it is important to develop and test new potentially effective treatments for depression and anxiety in order to treat a wider range of patients [15] and provide patients in the UK with choice alternatives.

### Morita Therapy

Morita Therapy is a psychotherapy developed in Japan by Dr Shoma Morita in 1919 [16] used for the treatment of common mental health problems. Morita Therapy was originally developed in inpatient settings for patients with particular psychological problems, including but not limited to GAD [17]. More recently, Morita Therapy has been applied to a wider range of conditions, including depression, and guidelines for practicing outpatient Morita Therapy have been developed [17]. Morita Therapy is now widely practiced in Japan, and has branches in various other countries including North America, Australia, China, Russia and Rwanda [18].

Morita Therapy is a holistic approach, aiming to improve functioning in everyday life, rather than an approach targeting specific symptoms [18]. The underlying premise is that unpleasant symptoms are part of the natural ecology of the human experience. Morita Therapy thus helps patients to re-orientate themselves in the natural world and takes a restorative approach to potentiate their natural healing capacity. Morita therapists help patients to move away from symptom preoccupation and combat, which it is conceptualised both interfere with this natural recovery process and lead to preoccupation with and worsening of symptoms [17]. By helping patients to accept that undesired symptoms are natural features of human emotion rather than something to control or eliminate, and that emotions ebb and flow as a matter of course and can be lived with, Morita Therapy is in sharp contrast to established western psychotherapeutic approaches with their focus on symptom elimination. In Morita Therapy, patients are taught to live with, rather than be without, unpleasant emotions.

### Uncertainties: The need for a mixed methods feasibility study

As with the development of many other treatments to date [15], initial evidence for Morita Therapy’s efficacy is largely based on case studies, predominantly conducted in Japan. A narrative review of forty-nine such studies and four quasi-experimental studies indicated that Morita Therapy has been reported as effective for a diverse range of issues, but that further work is required to both standardise its delivery and investigate its efficacy in controlled trials (personal communications: Minami, M).

Furthermore, Morita Therapy is currently little known in the UK. Thus, evidence of the efficacy of Morita Therapy based on truly experimental studies, and evidence of the effectiveness of Morita Therapy specifically for a UK population, has not yet been established. Whilst a fully-powered UK randomised controlled trial (RCT) of Morita Therapy versus treatment as usual is needed to establish the effects of Morita Therapy, a number of clinical, procedural and methodological uncertainties currently prevent us moving immediately to such a trial.

With respect to clinical uncertainties, the operationalisability of the UK Morita Therapy outpatient protocol, and the acceptability of both the protocol specifically and Morita Therapy in general, is unknown. Gathering data on these uncertainties is essential to ensure that the treatment administered in a large-scale trial is deliverable by therapists, and acceptable to both therapists and patients.

With respect to procedural uncertainties, information is required on the likely rates of recruitment to and retention in a trial of Morita Therapy, and of treatment adherence, in order to assess the feasibility of a trial and inform the required sample size. With respect to methodological uncertainties, estimates of the variance in participant outcomes and information on how these correlate with baseline scores are also required to inform future sample size calculations.

In line with the Medical Research Council (MRC) framework for the development and evaluation of complex interventions [19], all such uncertainties are appropriate to address within a pilot trial and feasibility study [20]. In order to both collect the required quantitative data and understand people’s views of Morita Therapy, qualitative work will be embedded in a pilot randomised controlled trial of Morita Therapy compared to treatment as usual, and merged with quantitative data on treatment adherence to potentially help explain variability in participants’ therapeutic engagement.

### Study purpose

The purpose of this study is to prepare the ground for the design and conduct of a fully-powered RCT of Morita Therapy plus treatment as usual versus treatment as usual alone, or to conclude that such a trial is not appropriate and/or feasible.

### Research questions

1. What proportion of participants approached to take part in the trial will agree to do so?
2. What proportion of participants who agree to take part in the trial will remain in the trial at four month follow-up?
3. What proportion of participants who agree to take part in Morita Therapy will adhere to a pre-defined per-protocol dose of Morita Therapy?
4. What is the variance in participant outcomes following Morita Therapy plus treatment as usual and treatment as usual alone, and how do they correlate with participants’ baseline scores?
5. What are the estimated between-group differences (and 95 % confidence intervals) in participant outcomes following Morita Therapy plus treatment as usual and treatment as usual alone?
6. How acceptable is Morita Therapy to participants and therapists?
7. How do participants’ views about Morita Therapy relate to the variability in the number of treatment sessions they attend?

### Criteria for success

The criteria to be met in order to deem a fully-powered RCT feasible as is [20] are:

1. A sufficient number of participants to populate a fully-powered trial are likely to be recruited and retained, i.e. we recruit at the rate anticipated in the pilot trial (12 % of those invited) and experience an attrition rate no higher than 20 % of those randomised, in line with our other National Institute of Health Research (NIHR) mental health trials [21–23]. We will consider whether protocol modification or close monitoring during a fully-powered RCT will address any failure to meet these criteria [20].
2. The levels of engagement with and adherence to Morita Therapy are likely to be on par with our other NIHR mental health trials [23], i.e. at least 65 % of patients allocated to Morita Therapy attend at least 40 % of treatment sessions. Any failure to meet this criterion will be considered in the light of participants’ views on the acceptability of Morita Therapy in order to determine whether protocol modification or close monitoring are sufficient to deem a fully-powered RCT feasible [20].
3. It is likely that a Morita Therapy outpatient protocol can be produced which is acceptable to patients and therapists, and deliverable by therapists, as defined by responses to qualitative interviewing.

## Methods/Design

### Study design

We will incorporate exploratory and explanatory components in a mixed methods embedded design [24]. Thus, we will embed semi-structured qualitative interviews within a pilot randomised controlled trial of Morita Therapy plus treatment as usual versus treatment as usual alone for people with depression, with or without anxiety disorders. We will give quantitative and qualitative components equal priority and mix them interactively at the design level within a program-objective framework [24]. For these two components, we will collect data concurrently and analyse data simultaneously. We will use quantitative data to assess the feasibility of trial recruitment, retention and treatment adherence, and to inform any future sample size calculations. We will collect qualitative data on participants’ and therapists’ views of Morita Therapy. By merging qualitative and quantitative data, we aim to explain variability in participants’ treatment adherence and develop a richer understanding of the feasibility, acceptability and appropriateness of Morita Therapy (Table 1).

### Philosophical assumptions

Our decision to use a mixed methods design is driven by the primary importance we give to addressing the uncertainties associated with running a fully-powered RCT. Thus, we are guided by a pragmatic philosophy: we prioritise our research objectives and the methods which will lead to the best evidence with regards to those objectives [25]. Consistent with a pragmatic worldview, we will also approach the objectives from a pluralistic perspective, combine deductive and inductive modes of reasoning, and allow for a singular view and multiple views of reality in how we come to understand and interpret our findings [25].

### Pilot Randomised Controlled Trial

#### Sample size

A conventional power calculation is inappropriate for the purpose of a pilot trial [20]. Instead, we have calculated the sample size in order to provide useful information about the aspects of the study being assessed for feasibility [20]. Thus, we have constructed confidence intervals based on certain criteria for success [20], specifically: recruiting at a rate of 12 % of those invited and experiencing an attrition rate no higher than 20 % of those randomised. We expect to invite a total of 570 participants to participate in the trial. Thus, we expect to recruit 72 participants into the trial, and follow-up 60 participants (30 in each trial arm).

Inviting 570 participants is sufficient to estimate a participation rate (as percentage of subjects invited) of 10 % with a margin of error of +/− 2.46 %, or to estimate a participation rate of 12 % with a margin of error of +/− 2.67 %, or to estimate a participation rate of 15 % with a margin of error of +/− 2.93 %, based on 95 % confidence intervals. Recruiting 72 participants is sufficient to estimate a follow-up rate (as percentage of participants randomised) of 80 % with a margin of error of +/− 9.24 %, or to estimate a follow-up rate of 85 % with a margin of error of +/− 8.25 %, based on 95 % confidence intervals.

In addition, we will calculate the standard deviation of participant outcomes and the correlation between baseline and four month follow-up scores, which can be used to refine future sample size calculations to incorporate the additional precision obtained from adjusting for baseline scores when comparing outcome scores between the trial arms. 30 participants in each group is sufficient to estimate: (i) the standard deviation of continuous outcomes to within 22 % of their true value based on the upper limit of the 95 % confidence interval; (ii) a Pearson’s correlation coefficient between baseline and follow-up scores with a margin of error of +/− 0.1 if the true correlation is 0.8, or with a margin of error of +/− .14 if the true correlation is 0.7, or with a margin of error of +/− 0.17 if the true correlation is 0.6.

30 participants per group is also in line with the general rule of thumb for using pilot studies to reliably estimate variance for participant outcomes [26]. With these factors in mind, we consider 60 participants at follow-up to be both sufficient to provide useful information and reasonable to recruit for within the constraints of our pilot trial and have, therefore, selected 72 as our target sample size, inflating our sample by 20 % to take account of predicted attrition.

#### Participant inclusion criteria

Eligible participants will be aged 18 or over with Diagnostic and Statistical Manual of Mental Disorders (DSM) Major Depressive Disorder, with or without accompanying DSM anxiety disorder(s).

#### Participant exclusion criteria

Given the exploratory nature of this trial (and any fully-powered evaluation), and thus the requirement for reasonable internal validity with a homogenous and tightly defined population, we will identify and exclude people who are cognitively impaired, have bipolar disorder or psychosis/psychotic symptoms, or are substance dependent. Cognitive impairment will be determined using the Mini-Cog, whereby a score of 0, or 1–2 with an abnormal clock-face, would indicate sufficient cognitive impairment to be excluded [27]. Bipolar disorder, psychosis and substance dependence will be established according to the DSM.

We will also exclude participants whose risk of suicide is sufficiently acute to demand immediate management by a specialist mental health crisis team, and those who are currently in receipt of psychological therapy. Psychological therapy includes any formal standard course of psychological (talking) therapy, such as Cognitive Behavioural Therapy. Ad hoc contact with a therapist or counsellor will not be considered to meet this exclusion criterion. Participants will be eligible regardless of whether they are in receipt of antidepressant medication or have received psychological therapy in the past.

#### Participant identification and recruitment

Our main method of recruitment will be through searches of General Practice records, conducted by Practice staff. We will recruit six GP Practices in Devon. All GP Practices who are able to access the University of Exeter’s Mood Disorders Centre (MDC) Accessing Evidence-Based Psychological Therapies (AccEPT) Clinic (those within the National Health Service Northern, Eastern and Western Devon Clinical Commissioning Group) will be eligible.

Practice record searches will be limited to patients aged 18 or over and seen within the past three months for depression. The resulting patient names will be screened by the GP with whom the patient is registered for any patients known to meet exclusion criteria or for whom the GP considers the trial unsuitable. The remaining patients will be sent invitations to participate in the trial by Practice staff.

We will also place adverts on websites of the University of Exeter Medical School and AccEPT Clinic, place leaflets in the waiting rooms of consenting Devon General Practices and circulate an email invitation to former MDC participants who have consented to such contact. All invitations and adverts will include a study summary sheet [see Additional file 1] and permission to contact form [see Additional file 2] (Figs. 1 and 2).

#### Screening and baseline

We will telephone all people who return their permission to contact form to the study team to assess possible eligibility using a standard two-question case-finding instrument for depression [28] and arrange baseline interviews with potentially eligible and willing participants who will be sent a confirmation letter and full participant information leaflet [see Additional file 3]. We will hold baseline interviews at University of Exeter premises or the participant’s home, depending on participant preference. At interview, we will explain the study in full and assess eligibility according to the Mini-Cog [27] (to screen for cognitive impairment) and standard clinical interview (Structured Clinical Interview for DSM-IV-TR Axis I Disorders, Clinical Trials Version [29]). If eligible and once fully informed, participants will be asked to complete a consent form [see Additional file 4] and entered into the trial. Ineligible participants will be returned to the care of their GP.

#### Randomisation, allocation concealment and blinding

We will allocate participants in a 1:1 ratio to either Morita Therapy plus treatment as usual or treatment as usual alone, stratified according to their symptom severity on the nine item version of the Patient Health Questionnaire (PHQ-9) [30], specifically whether they score below 19 or 19 and above, given that a score of 19 is the median score of depressed participants in our previous research [21, 23]. Allocation will be minimised to maximise the likelihood of balance in the stratification variable across the two trial arms. To ensure allocation concealment, we will undertake randomisation through the use of an externally administered, password-protected randomisation website independently developed and maintained by the Exeter Clinical Trials Unit.

The researchers will not be blinded to allocation due to the different pathways to be followed for each trial arm. Baseline and follow-up data will be self-reported and the risk of bias related to lack of blinding will be both minimal and tolerable.

#### Trial interventions

##### Morita Therapy plus treatment as usual

We will ask participants in the Morita Therapy plus treatment as usual trial arm not to engage in other formal courses of psychological therapy elsewhere during the course of their treatment. Otherwise, these participants will be free to access any other usual care and medication in liaison with their GP.

Morita Therapy will consist of eight to twelve one hour face-to-face weekly sessions and be delivered at the University of Exeter’s MDC AccEPT clinic [31] by two research therapists trained in Morita Therapy and experienced in both the delivery of complex psychological interventions and adopting different modes of treatment, including experimental treatments. Therapist training took place over 6 months and included background reading, attending presentations, involvement in the development and review of the UK Morita Therapy outpatient protocol, and practical training led by DAR, a clinically qualified academic and 10 year member of the Japanese Society for Morita Therapy. Practical training was experiential, involving role plays, diary examples, additional reading and peer support. The therapists are not accredited as there is no accreditation process for Morita Therapy within the UK.

Therapists will follow the UK Morita Therapy outpatient protocol developed by the study researchers from multiple sources of literature on the delivery and practice of Morita Therapy [16–18, 32–35] and by considering the views of potential participants and therapists about Morita Therapy, as explored in qualitative interviews, in order to enhance the suitability of Morita Therapy for a UK population. DAR will provide fortnightly supervision of cases together with advice and support. A qualitative checklist highlighting the key components of Morita Therapy will be used as an aide memoir to structure supervision discussions and the assessment of adherence and fidelity. With the patient’s consent, all therapy sessions will be audio recorded. We will use the first two recordings for each therapist to confirm their adherence to the Morita Therapy outpatient protocol and a further 10 %, stratified by length of time in treatment, to evaluate fidelity to the protocol, which will inform therapist supervision.

During therapy, patients will progress through four stages of rest and increasing action taking in order to address fatigue, expand peripheral attention and move from a mood-oriented to purpose-oriented and action-based lifestyle. Therapists will aid patients in re-appraising their symptoms as part of the natural ecology of human experience; recognising the vicious cycle of symptom aggravation created by fixation on symptoms, contradictions between reality and the ideal, and attempts to fight or control otherwise inevitable emotions; and moving from a position of preoccupation with symptoms to the acceptance of spontaneous affective experiences. Therapists will continually reinforce the patient’s shift from self-reflection towards a focus on constructive action and the external environment. Throughout therapy, patients will also complete a daily diary for therapists to comment on, to increase communication and the opportunity for therapist reinforcement.

#### Treatment as usual alone

We have selected treatment as usual as our trial comparator as a reflection of the trial comparator which would be selected for a fully-powered RCT, in which our key interest would be whether Morita Therapy plus treatment as usual has superior or equivalent effectiveness to current clinical practice in the UK, in which people have access to GP care and a range of other treatments. Thus, a large scale RCT would be a pragmatic trial embedded within the healthcare environment in which Morita Therapy would be delivered, seeking to establish whether Morita Therapy could be useful in addition to the options currently available to depressed patients in the UK.

Thus, in this pilot trial we will replicate ‘treatment as usual’ by making no specific patient-level recommendation or requirement to alter the usual treatment received by depressed patients in the UK, and the study will not place any restrictions on the treatment options available to these participants. GPs will treat and refer participants as would be their normal practice and participants in this trial arm are free to access any other care and services, including formal courses of psychological therapy such as Cognitive Behavioural Therapy. All participants, irrespective of their allocation, are free to choose whether they take antidepressant medication or not. We will record the treatments received in the course of participants’ treatment as usual.

#### Outcomes

Given this is a feasibility study with a range of different aims, there is no single primary outcome measure. Rather, we will collect a variety of data at baseline interview and four months post-randomisation: severity of depressive symptoms (PHQ-9 [30]), severity of generalised anxiety symptoms (seven item Generalised Anxiety Disorder questionnaire: GAD-7 [36]), quality of life (Short Form 36 Health Survey Questionnaire: SF-36 [37]; Work and Social Adjustment Scale: WSAS [38]), and attitudes (The Morita Attitudinal Scale for Arugamama: MASA [39]). At four months post-randomisation, we anticipate that treatment for participants in the Morita Therapy plus treatment as usual trial arm will be complete. We will hold follow-ups at University of Exeter premises or the participant’s home, depending on participant preference, and apply all research measures to both groups of participants equally.

We will also collect data on the flow of participants through the trial. For participants in the Morita Therapy plus treatment as usual trial arm, therapists will also inform the researcher of the number of therapy sessions attended and the reason for ending treatment. We will not conduct an economic evaluation as part of this pilot trial, although at follow-up we will incorporate methods for collecting data on participants’ use of health and social care services as used in our recent mental health trials [23] (whereby we will establish the rates and nature of hospital visits; use of community, social and complementary services; and use of psychotropic medication since baseline assessment), in order to characterise treatment as usual and calculate the cost of each trial arm for a large-scale RCT.

### Semi-structured Interviews

#### Sample and setting

We will invite all participants who are allocated to Morita Therapy plus treatment as usual for a post-treatment semi-structured interview, thus selecting as diverse a sample as possible within this pilot trial. This will provide a maximum of 30 participants (all those retained in the Morita Therapy trial arm). We will also invite the two therapists providing Morita Therapy to interview. We will hold participant interviews at University of Exeter premises or the participant’s home, depending on participant preference. Therapist interviews will be conducted at the AccEPT Clinic.

#### Recruitment

We will explain the purpose and content of the interview to participants in the participant information leaflet, and determine their consent to participate at baseline interview. We will send therapists an interviewee information leaflet explaining the interview prior to a pre-trial meeting, and establish their consent to participate during this meeting. Upon completion of Morita Therapy (delivery, for therapists), we will contact participants to establish whether they are still willing to be interviewed, remind them of what will be involved and answer any questions. For willing participants, we will arrange an interview no sooner than 48 hours later and send an interview confirmation letter explaining the opportunity to rearrange or cancel the interview at any time.

#### Interview process and questions

We will undertake semi-structured interviews to allow participants to describe their views of Morita Therapy. This method will enable us to investigate the meaning of participants’ responses, both exploring views on our predefined topics of interest and eliciting more detail on any emerging themes [40]. Interviews are expected to last up to one hour and will be audio-recorded with the participant’s consent. The interviewer will also take field notes during and after the interview.

We will follow topic guides established on the basis of our recent mental health trials addressing similar research questions [21, 23, 41] (which ask about participants’ views and experiences of treatment, any barriers to treatment, and the impact of treatment) and existing Morita Therapy literature. To explore the acceptability of Morita Therapy, we will ask participants to describe their understanding of Morita Therapy, explore their views and experiences of Morita Therapy and investigate potential barriers to/facilitating factors in engaging with Morita Therapy. In particular, we will explore participant’s views and experiences of the defining features of Morita Therapy in practice, such as the four stages and daily diaries. To explore the feasibility and appropriateness of our trial procedures, we will explore participants’ views on the support provided throughout the trial; procedures for recruitment, monitoring and data collection; and use of the MASA questionnaire. We aim to identify both procedures that facilitated the efficient running of the trial and any considered problematic.

### Analysis

We will first analyse the quantitative and qualitative data separately before integrating both types of information in a mixed methods analysis.

#### Quantitative analysis

Following double data entry into STATA v.11 [42], we will analyse recruitment, retention, treatment adherence and estimates of the participant-related data to inform the feasibility of and sample size calculation for a fully-powered trial. Thus, we will emphasise quantification and estimation rather than hypothesis testing. All analyses will be on an intention to treat basis and we will not impute missing data, although we will report outcome data that are missing in each trial arm and the reasons for missing data where possible.

We will use count data with calculated estimated margins of error, expressed as a percentage of both the total number of participants invited and in relation to the preceding step in recruitment, to quantify the flow of the participants through the trial. For each trial arm, we will quantify the number of participants who withdrew, could not be contacted or did not provide follow-up data for another reason. We will also express data as a percentage of the total number of participants in each trial arm. We will follow CONSORT guidelines, including the forthcoming pilot and feasibility extension [43], in reporting all data including the number of participants exiting the trial at each step and from whom we are unable to collect follow-up data. Descriptive statistics will also be used to describe the number of Morita Therapy sessions attended by participants allocated to Morita Therapy plus treatment as usual.

To measure the variance in participant outcomes, we will estimate the standard deviation around the mean PHQ-9, GAD-7, SF-36, WSAS and MASA scores at baseline and four months for both groups. We will also estimate the correlation between participants’ scores on these measures at baseline and at four months, which can be used to refine the sample size calculation for any fully-powered evaluation. Although we do not have the power to make inferential statements on between (or within) group differences and as such no p values will be calculated, we will also calculate and report the observed differences between Morita Therapy plus treatment as usual and treatment as usual alone on the mean changes in these measures from baseline to four month follow-up, and the 95 % confidence intervals around these figures.

#### Qualitative analysis

With participants’ permission, we will record and transcribe interviews verbatim. We will use NVivo10 [44] to organise the data and conduct a systematic analysis of interviews and field notes, using Framework analysis [45] to allow for the combination of both inductive and deductive approaches in the development of analytic categories. In line with this, an initial thematic framework will be developed as preliminary analysis is undertaken and subsequently as batches of transcriptions are analysed, iteratively combining our topic guide and the overall impression of the narratives in context. Using this framework, transcripts will be coded at the level of individual participants and then analysed thematically across the whole dataset as well as in the context of each participant’s interview using a constant comparison approach [46], whereby each piece of data (e.g. one statement or one theme) is compared with others for similarities and differences [47]. As we formulate explanations in this way, negative cases will be explored and explanations of variance provided [48], thus incorporating all observations relevant to our research question. Data collection and analysis will be iterative: we will amend our interviewing style to respond to emerging themes and explore deviant cases further in subsequent interviews as appropriate.

#### Mixed methods analysis

Our mixed methods analysis will be guided by both the nature of the quantitative and qualitative data that we ultimately obtain and the inferences that arise from our separate analysis of each [41]. Thus, the analysis we eventually undertake may differ to the analysis we propose [41]. Analytical techniques have been proposed below based on the methods summarised by Creswell and Plano Clark [24].

To explore how the qualitative data on the acceptability of Morita Therapy explains the quantitative findings on treatment adherence, we will merge these two types of data. Firstly, we will develop typologies of participants’ different views on the acceptability of Morita Therapy from the qualitative data, and for each typology we will present data on treatment adherence for participants to whom the typology applies [41]. Alongside this, we will also present data on fidelity to the therapy protocol if the qualitative data relates to particular sections of the protocol or stages of therapy. This will allow us to explore whether any issues with the acceptability of Morita Therapy relate to the treatment itself or the therapists’ delivery of treatment and thus aid us in identifying any ‘fatal flaws’ [49] of Morita Therapy requiring refinement in the future. Secondly, we will identify categories of participants defined by their treatment adherence and explore similar and different views on acceptability within and between categories [41].

We will consider the use of joint displays to summarise the quantitative data in relation to the qualitative themes for both of these purposes [41]. We will also integrate data on acceptability and treatment adherence in a case-oriented merged analysis display that will position cases (participants) on a scale of treatment adherence along with their qualitative data on acceptability [41].

### Ethical issues

We will conduct this trial in such a way as to protect the human rights and dignity of the participants, as reflected in the Helsinki Declaration [50]. The study has received ethical approval from the National Research Ethics Service South West – Frenchay (reference 15/SW/0103) and governance assurance from the National Health Service Research and Development Directorate (reference CG/JL), and has been approved by the University of Exeter Medical School following independent peer review.

Participants will not receive any financial inducement to participate. We will conform to Good Clinical Practice Guidelines, data protection and freedom of information acts. All data will be stored securely and anonymised wherever possible. All identifiable participant information will be stored separately to questionnaire data which will be coded by a trial ID number only. No published material will contain identifiable participant information.

#### Informed consent and withdrawal

The study researchers will be fully trained and supervised by senior academic and clinically qualified staff. All our information leaflets and consent forms have been produced using the current Health Research Authority’s online guidance for writing such documents [51], and are based on similar materials used in our other mental health trials as informed by Patient and Public Involvement.

Informed consent will be determined by a two phase process. Potential participants will receive a study summary sheet and a form on which to complete their contact details and confirm their permission for a researcher to contact them. We will telephone those who return this form to us, to assess their potential eligibility and answer any questions. For those who are eligible and willing, we will send a participant information leaflet and arrange a baseline interview at least 48 hours later, to allow the participant time to reflect on their decision to participate and change their mind if they so wish. Full informed consent will only be obtained at this interview where the information leaflet will be fully explained and the opportunity to ask questions given.

Consent to participate in the qualitative interview is optional; participants may participate in the pilot RCT only. We will explain the purpose and content of the interview in the participant information leaflet (or interviewee information sheet, for therapists), and note that a decision not to be interviewed will not affect participation in the trial. At baseline interview (for participants) and the pre-trial meeting (for therapists), we will answer any questions, explain the opportunity to stop and/or withdraw from the interview at any time and clarify steps to maintain confidentiality. We will ask willing participants to indicate their decision on a consent from. Consent for audio recording of the interview and/or therapy sessions is also optional.

We will treat informed consent as an ongoing process whereby participants may withdraw their consent to participate at any time, and set up communication and recording systems to enable us to monitor and act on such wishes. When obtaining consent, we will advise participants of this fact and that they may be asked to give a reason for their withdrawal but will not have to provide one. Participants allocated to Morita Therapy plus treatment as usual may withdraw from therapy and continue their involvement in the trial through participation in the follow-up and qualitative interview if they wish.

Should it come to our attention that a participant loses capacity to consent during the study according to the Mental Capacity Act 2005 [52], we will withdraw them from the study as per information provided to participants in the participant information leaflet. Within this leaflet, we will also inform participants that if they should withdraw or be withdrawn from the study, we will retain any data already provided to be used confidentially in relation to the purpose for which consent was sought.

#### Anticipated risks and benefits

No treatment will be withheld from participants taking part in this trial. All participants will remain under the care of their GP and will have access to primary care services in the usual way. Participants allocated to treatment as usual alone will be returned to the care of their GP with no restrictions placed on treatment options. Participants allocated to Morita Therapy plus treatment as usual will be asked not to engage in other formal courses of psychological therapy during their treatment, as it is not considered good practice to engage in more than one psychological therapy at once. Should participants in this trial arm wish to engage in other psychological therapy elsewhere, a discussion will be held with their therapist to establish which therapy option is in the participant’s best interests.

Participants allocated to Morita Therapy plus treatment as usual will take part in an alternative therapeutic approach to psychopathology which is widely practiced in Japan and somewhat elsewhere. Morita Therapy has been practiced since the 1920s and is not known to be associated with any risks to patients. It is possible that participation in therapy focused on psychopathology may cause distress to some participants, however participants in the Morita Therapy trial arm will receive an intensive level of monitoring so that any worsening or at suicidal risk will be identified and directed to appropriate care. Similarly, we will address any impact of potentially distressing questions within our assessment and outcome measures by following our protocols for responding to risk and directing participants to appropriate care. Additionally, we will report any serious adverse events reported to a therapist or researcher which are thought to be treatment related to the trial sponsor, Research Ethics Committee and independent oversight clinician (see section on study oversight).

The patient information leaflet will explain that participants allocated to Morita Therapy plus treatment as usual will no longer be offered such therapy once they have received a full “dose” (up to twelve sessions), but will be referred back to their GP with whom they could consider access to other treatments. We will ensure participants are reminded of these factors throughout the trial.

The University of Exeter has insurance to cover the potential legal liability for any harm to participants arising from the management of this trial. We will also provide potential participants with information about the possible benefits and risks of taking part in the trial in the participant information leaflet, and give them the opportunity to discuss this issue with us before consenting. We will inform participants in writing if new information comes to light which may affect their willingness to participate in the trial.

#### Managing risk of suicide

Inherent in the nature of the population under scrutiny is the risk of suicide. We will follow good clinical practice in monitoring for suicide risk during all appointments and explain to participants that we will contact their GP or specialist if deemed necessary in line with our risk protocol. If an acute risk is present, we will seek advice from the participant’s GP (or the duty GP) immediately and/or follow locally established suicide management plans. All clinicians and researchers will be familiar with established risk protocols used in our previous research trials and/or within the AccEPT Clinic, specifically trained in risk assessment and supervised by experienced clinicians. We will put in place systems to ensure that senior academic and clinically qualified staff are notified should there be any risk to a participant’s safety.

### Patient and public involvement

We have developed the patient materials on the basis of both consultation with a Public and Patient Involvement Expert and similar materials used in our other mental health trials which received feedback from Public and Patient Involvement groups such as the National Institute for Health Research Collaboration for Leadership in Applied Health Research and Care (CLAHRC) South West Peninsula (PenCLAHRC) [53] Patient and Public Involvement Group (PenPIG). This feedback has helped us to ensure that our research respects the rights, safety and dignity of participants. Ensuring that our research materials are sensitive and consistent with the views of people with depression will also aid us in recruitment and participants’ engagement in and openness during interviews.

Following completion of the pilot trial, to ensure that our results reach our former trial participants and people with mental health issues in a way that is meaningful and accessible, we will establish an advisory group comprising members of PenPIG and follow national good practice guidance for researchers on public involvement in research and the paying of representatives [53]. The group will be involved in the dissemination of the results to the public and patients using accessible channels and their own conference and group meetings. Training in presentation skills will be arranged for members of the group should they consider this helpful. We will also consult the advisory group on the development of a summary sheet explaining the results of the study and their implications in lay terms, to be sent to consenting former trial participants.

### Dissemination protocol

In addition to the above details on the dissemination of results to the public and former trial participants, we will disseminate the results of this study in a full internal report and intend to publish our results in a peer reviewed scientific journal. Authors will be those considered to have made a substantive intellectual contribution to the study. The main output from this study will be the information required to design and seek funding to conduct a definitive trial of Morita Therapy. Thus, in the long term we aim to contribute to national guidelines for the treatment of depression and anxiety.

The investigators and relevant authorities will have access to the trial dataset. Furthermore, we will store anonymised research data and outputs in the University of Exeter’s Open Research Exeter repository [54] in order to facilitate open access to, and the impact of, our research.

### Study oversight

This research forms part of the first author and Chief Investigator’s (HVRS) PhD programme of studies for which she is supervised by DAR and JF. Trial conduct will be discussed between the Chief Investigator and her supervisors at monthly supervision meetings.

Although the convention of a formal Data Monitoring and Ethics Committee is not appropriate for the scale of this study, an independent clinician will act in this capacity in order to review serious adverse events which are thought to be treatment related, and any substantive protocol amendments. All such amendments will be communicated to the relevant authorities as deemed necessary.

#### Forecast execution dates

The preparatory period started in October 2014. Recruitment is running from September 2015 for approximately ten months. Follow-up and qualitative data will be collected from January 2016 to November 2016. Data analysis and reporting are expected to take another nine months. The total duration of the study will be 24 months.

## Discussion

By preparing the ground for the design and conduct of a large-scale RCT, this study will contribute important information towards the development and subsequent evaluation of Morita Therapy for the treatment of depression and anxiety for the first time in the UK. One strength of our study design is that the proposed methods are appropriate for undertaking a feasibility study [41]. Our study purpose and research questions are in line with the National Institute for Health Research Trials and Studies’ definition of a feasibility study [55] endorsed by Arain and colleagues [56]. We have calculated the RCT sample size based on the key feasibility objectives around recruitment and retention rates, and will calculate the variance in participant outcomes and their correlation with baseline scores to inform future sample size calculations. We will also calculate the observed differences between Morita Therapy plus treatment as usual and treatment as usual alone on the mean changes in outcome measures, although we will not make inferential statements or evaluate these outcomes. Rather than identifying a primary outcome measure, we have designed both the pilot trial and qualitative interviews to allow us to test the uncertainties associated with designing and running a large-scale fully-powered RCT of Morita Therapy plus treatment as usual versus treatment as usual alone.

To embrace the complexity of developing and evaluating interventions and provide a comprehensive understanding of the intervention in question, no one method will suffice [25]. Thus, a further strength of this study is our explicit commitment to a mixed methods approach and transparent description of the way in which quantitative and qualitative components will be integrated [41, 57]. We have carefully considered guidance on maximising the impact of qualitative research in feasibility studies [49] and described our proposal in line with recommendations for Good Reporting of a Mixed Methods Study [57], which we will continue to follow in our future reporting. Our embedded mixed methods design reflects key decisions we have reached on the levels of interaction, priority, timing and procedures in the mixing of the quantitative and qualitative components [24, 41]. Thus, we will interactively mix the two components before final interpretation, at both the design and analysis levels, by embedding qualitative interviews within the pilot RCT in a program-objective framework; give the two components equal priority; undertake the pilot trial and qualitative interviews concurrently; and analyse data from the two components simultaneously.

We have specified research question seven to frame the integration of results from the quantitative and qualitative strands, to help explain variability in treatment adherence and thus facilitate a more complex picture of the acceptability of Morita Therapy [24]. By qualitatively exploring the acceptability of both Morita Therapy and our trial procedures, and integrating the qualitative and quantitative data, we will facilitate both the interpretation of our pilot trial findings and the feasibility and/or efficiency of any large-scale RCT, thus allowing us to optimise both our intervention and trial conduct in the future [58]. The integration of quantitative and qualitative methods will enable us to address both exploratory and explanatory research questions simultaneously, and help to reduce the limitations of each individual method whilst retaining their strengths [25]. Ultimately, by implementing an embedded mixed methods design, this study will better prepare the ground for a large-scale fully-powered RCT of Morita Therapy plus treatment as usual versus treatment as usual alone than would be possible from either a quantitative or qualitative approach alone [25, 41].

### Trial status

Recruitment commenced in September 2015 and is ongoing.

## Additional files

Additional file 1: Study Summary Sheet. (PDF 222 kb) Additional file 2: Permission to contact form. (PDF 160 kb) Additional file 3: Participant Information Leaflet. (PDF 766 kb) Additional file 4: Model Consent Form. (PDF 246 kb)

## Abbreviations

GAD: Generalised Anxiety Disorder
GPs: General Practitioners
NICE: National Institute for Health and Care Excellence
IAPT: Improving Access to Psychological Therapies
RCT: Randomised Controlled Trial
MRC: Medical Research Council
NIHR: National Institute for Health Research
DSM: Diagnostic and Statistical Manual of Mental Disorders
MDC: Mood Disorders Centre
AccEPT: Accessing Evidence Based Psychological Therapies
PHQ-9: nine item version of the Patient Health Questionnaire
GAD-7: seven item Generalised Anxiety Disorder questionnaire
SF-36: Short Form 36 Health Survey Questionnaire
WSAS: Work and Social Adjustment Scale
MASA: The Morita Attitudinal Scale for Arugamama
CLAHRC: National Institute for Health Research Collaboration for Leadership in Applied Health Research and Care
PenCLAHRC: National Institute for Health Research Collaboration for Leadership in Applied Health Research and Care South West Peninsula
PenPIG: National Institute for Health Research Collaboration for Leadership in Applied Health Research and Care South West Peninsula Patient and Public Involvement Group
