# Supplementary material for: Morita therapy for depression and anxiety (Morita Trial): study protocol for a pilot randomised controlled trial
Source: Trials. 2016 Mar 24;17:161. doi: 10.1186/s13063-016-1279-3 (PMC4806496; doi:10.1186/s13063-016-1279-3)
Supplement: Additional file 1: — Study Summary Sheet. (PDF 222 kb) [file 13063_2016_1279_MOESM1_ESM.pdf]

Morita

The Morita Therapy Trial

Patient Summary Information Leaflet

The Morita Trial (Morita Therapy for Depression and Anxiety: A Feasibility and Pilot Study)

This is a very short summary of our study. **It asks you to consider taking part in our research and for your permission for a researcher to contact you about the study.**

Introduction. We are carrying out a study at the University of Exeter Medical School to help develop a large trial of Morita therapy, a new treatment for depression and anxiety. We are writing to you because your GP surgery has agreed to help us with this by sending information to you after you visited your GP reporting symptoms that are experienced by many people with depression.

What is the treatment that is being tested? This study will provide us with the information we need to investigate the effects of Morita therapy. Morita therapy is a treatment for mental health problems which is widely practiced in Japan but little known in the UK. Ultimately, we would like to carry out a large trial to find out whether Morita therapy is effective for people here who experience depression and anxiety. Before we can do this, we need to carry out a small trial to develop the treatment and test our procedures. We also want to know if Morita therapy is acceptable to patients and to clinicians.

What will happen if I take part? If you would like to take part, a researcher will speak with you to see if you are eligible for the study and to explain it in more detail. If you are eligible and agree to take part you will receive either Morita therapy or the usual care which is available to you from your GP and elsewhere.

This study is a randomised controlled trial which means that the decision about whether you would receive Morita therapy or not is made completely by chance. Half of our participants will receive Morita therapy and half will receive usual care. We will meet with

everyone who would like to take part to find out if they are eligible and to fill in some questionnaires. Four months later, we will also meet again with everyone who takes part in the study to fill in the questionnaires again.

Morita therapy will involve between eight and twelve face to face sessions over eight to twelve weeks, a one hour session per week. The treatment will be delivered by a trained therapist at the University of Exeter's AccEPT Clinic. Everyone who receives Morita therapy will also be invited to an interview after they have completed treatment to find out their views of Morita therapy and their experiences of taking part in the trial, if they would like to.

At the end of the trial, we will look at how many people took part and filled in our questionnaires. We will also use the information people provide to work out how big a large trial needs to be, and to see whether Morita therapy is acceptable to patients and therapists.

Will my taking part in this study be kept confidential? We will keep all of the information that we collect about you during the course of the research strictly confidential within the study team.

What should I do now? If you are interested in the study and are happy for a researcher to contact you to discuss whether or not you would like to take part, you should **complete the enclosed 'Permission for Researcher to Contact' form and return it to the study researcher in the freepost envelope enclosed.** Someone working on the study will then contact you with more information about this study and arrange a time to meet you and answer any questions you may have.

Meanwhile, if you would like to find out more, you can contact the Morita Trial researcher Holly on 01392 727412 or at h.v.s.sugg@exeter.ac.uk.

Thank you for reading this and for considering taking part in this study.

UNIVERSITY OF EXETER | MEDICAL SCHOOL
