# Supplementary material for: Morita therapy for depression and anxiety (Morita Trial): study protocol for a pilot randomised controlled trial
Source: Trials. 2016 Mar 24;17:161. doi: 10.1186/s13063-016-1279-3 (PMC4806496; doi:10.1186/s13063-016-1279-3)
Supplement: Additional file 2: — Permission to contact form. (PDF 160 kb) [file 13063_2016_1279_MOESM2_ESM.pdf]

‘Permission for Researcher to Contact’ Form

The Morita Trial (Morita Therapy for Depression and Anxiety: A Feasibility and Pilot Study)

I confirm that I have read and understand the summary sheet for the above study and I am happy for a researcher to contact me to discuss whether or not I would like to take part.
I understand that my participation is voluntary and that I am free to withdraw at any time without giving any reason, without my medical care or legal rights being affected.

Name...
(Please print name)

Address...

....

....

Signature...

Name of GP and Surgery:

Telephone contact details:

Day... May we leave a voice mail message? Y / N

Evening... May we leave a voice mail message? Y / N

Mobile... May we leave a voice mail message? Y / N

Email address...

Please return to:

Holly Sugg, Morita Trial Study Researcher
University of Exeter Medical School
Room 1.33, South Cloisters
St Luke's Campus
Heavitree Road
Exeter EX1 2LU

Email: h.v.s.sugg@exeter.ac.uk Office telephone: 01392 727412
