# Supplementary material for: Morita therapy for depression and anxiety (Morita Trial): study protocol for a pilot randomised controlled trial
Source: Trials. 2016 Mar 24;17:161. doi: 10.1186/s13063-016-1279-3 (PMC4806496; doi:10.1186/s13063-016-1279-3)
Supplement: Additional file 3: — Participant Information Leaflet. (PDF 766 kb) [file 13063_2016_1279_MOESM3_ESM.pdf]

Participant Information Leaflet

The Morita Trial (Morita Therapy for Depression and Anxiety: A Feasibility and Pilot Study)

Thank you for returning your permission to contact form for this research study. Before you decide whether you want to take part or not it is important for you to understand why the research is being done and what it will involve. Please take time to read the following information carefully and discuss it with others if you wish. Ask us if there is anything that is not clear or if you would like more information. Take time to decide whether or not you wish to take part.

What is the purpose of the study?

Depression and anxiety cause misery to many people and are major health problems in the UK. Although some current treatments are effective for some people, they do not work for everybody and it is important to develop new therapies so as to offer people a choice of treatments which may suit them. One possible treatment for depression and anxiety is called Morita therapy. Although this treatment is widely used in Japan, we do not know if it is effective for and acceptable to patients and clinicians in the UK. By carrying out a large clinical trial to assess the outcomes of Morita therapy, we hope to find out whether it is an effective depression and anxiety treatment for people here. However, before we can do this, we need to test the treatment and our procedures in a small trial. We also need to speak with patients and therapists to find out what they think about Morita therapy.

Why have I been invited and will the study be suitable for me?

Either your GP surgery or local Improving Access to Psychological Therapies (IAPT) service is taking part in this trial and sent you a letter asking you to consider taking part because you reported symptoms that are experienced by many people with depression which we are treating in this study, or you have contacted us in response to one of our adverts for the study online or in your GP waiting room. This information sheet is for you to keep, if you

decide to take part one of our research team will go through the information sheet with you and answer any questions you have. You will also be asked some questions by the researcher to see if you are eligible to be included in the study. You may take part whether you are taking medication or not, and whether you have tried therapy in the past or not. However, if you are currently receiving another psychological therapy you will not be able to take part in this study.

Do I have to take part?

No. It is entirely up to you to decide whether or not to take part. If you do decide to take part you will be asked to sign a consent form. If you do decide to take part, you will still be still free to withdraw at any time and without giving a reason. A decision to withdraw or not to take part will not affect the care you receive in any way.

What is being tested?

We are running a small clinical trial to compare Morita therapy for depression and anxiety with usual care. We are intending to carry out a large trial to test Morita therapy but before we can do this, we need to find out how big such a trial needs to be and how many people we need to approach to take part. We also need to know what patients and therapists think about Morita therapy. A small trial will allow us to develop the treatment and our trial methods, and we will use qualitative interviews to find out if Morita therapy is acceptable to people.

Morita therapy is based on the idea that symptoms of depression and anxiety are a natural part of peoples' experience, but that responses to these feelings can make them worse. In particular, focusing too much on trying to change unpleasant feelings can actually fuel them, like being caught in a vicious cycle. The therapist helps you to understand these behaviours and how they can be unhelpful. As opposed to current therapies such as Cognitive Behavioural therapy, the aim is therefore to focus on how to live constructively in spite of symptoms, rather than focusing on changing thoughts and feelings.

Usual care means you will not receive any treatment through the study itself but there will be no restrictions placed on the care or treatment you may wish to access elsewhere. You will be returned to the care of your GP and may discuss treatment options with them.

What will happen if I take part?

Thank you for speaking with us over the phone and arranging to meet with us to find out if you are eligible to take part in the study. You can ask us about the study at any time. If we confirm at our meeting that you are eligible and you agree to take part you will receive Morita therapy or usual care. However, if after you have spoken with the researcher and answered some questions it is found that you are not eligible to take part, we are really sorry if it causes you disappointment and thank you for your interest and time that you have given. If you are not eligible to take part we would refer you back to your GP to continue treatment in the normal way.

If you are eligible to take part we need to explain that this study is a randomised controlled trial which means that once you have been interviewed by a researcher and have decided you would like to take part, the decision about whether you receive Morita therapy or usual care is made completely by chance. In this trial half of our participants will receive Morita therapy and half will receive usual care. We will allocate you to either Morita therapy or usual care by assigning you a personal identification number, known only to the research team, which will be entered into a secure computer system that picks the numbers at random and allocates them to one of the options at random. We will let your GP know that you are participating in this study.

If you are allocated to Morita therapy, you will receive between eight and twelve sessions of one hour duration with a trained therapist once a week, spread over eight to twelve weeks. The therapist will see you face to face and help you to complete a daily diary between sessions which outlines your daily activities. If you are allocated to usual care, we will let your GP know and you will be free to access any other treatments which you can discuss with your GP.

Once you have been allocated to Morita therapy or usual care, you may be invited to a more in-depth interview about why you have chosen to take part in the trial. We will also meet you again for a follow-up appointment with a researcher four months after our first meeting, to complete a number of questionnaires. If you are allocated to Morita therapy, once you have attended all the treatment sessions we will also invite you to take part in a more in-depth interview about your views of Morita therapy and experiences of taking part in the trial. Overall, your involvement in the study will be for a maximum of five months although the research study will last for two years.

What information do you need from me?

At our arranged meeting we will find out more about you. We will need to ask about your current and past mental health as well as your life more generally. We will ask you some questions about how you have been feeling recently and there will be a few questionnaires that we would like you to fill out. You will also be able to ask any questions you may have about the study. This meeting will take about two hours. We expect that the follow-up appointment will take no more than around one hour and we will collect some more questionnaires from you at this appointment.

We are interested in finding out about why people have volunteered to take part in the trial. Therefore, we may ask you to attend a more in-depth interview of up to one hour after we have seen you for our first meeting. We are also interested in finding out what people think of Morita therapy and their experiences of taking part in the trial. Therefore, we will ask people who are allocated to receive Morita therapy to attend a more in-depth interview of around one hour after they have completed treatment. We would like to audio record these interviews if you are happy for us to. The interviews would be conducted over the phone or face to face at your home. Alternatively, if you cannot speak on the phone or meet a study researcher at your home, we will arrange to meet with you at the University of Exeter. There is a separate part to the consent form to allow you to give your consent for these interviews, and you do not have to agree to it if you do not want to. If you choose not to take part in the interviews, you can still take part in the trial and it will not affect the standard of care you receive. If you agree, the recordings will be anonymously transcribed (typed up word for word, with any information which may identify you or your family or friends removed) before being destroyed.

Will I have to do anything differently?

No, there are no restrictions in your lifestyle from taking part in this research. You should continue to follow the advice of your GP if they remain involved in your care.

Will I be paid to take part?

No. We cannot pay people to attend appointments with their therapist and we will not reimburse travel expenses for these. Occasionally, it may be necessary for people to attend additional interviews with a study researcher at the University of Exeter for which we will pay travel expenses.

What happens when the research study stops?

We will encourage you to continue to see your GP who will treat you as s/he feels is best for you and with your agreement.

Are there any side effects, disadvantages and risks of taking part?

We are not aware of any side effects, disadvantages or risks to you of taking part in this research. If any relevant new information comes to light which may affect you or your decision to take part in the trial we will inform you of this.

What are the possible benefits of taking part?

Many people in Japan and other countries have found Morita therapy helpful and it has been shown to have a positive effect for some people with mental health problems such as depression and anxiety. If you are allocated to receive Morita therapy, we hope that the treatment you are given will help you. However, we cannot guarantee that you will benefit from the treatment. The information we get from this study may help us to treat future patients with depression and anxiety better.

What will happen to the results of this study?

We will send you a summary of the results of the study if you would like us to. We intend to publish the results of this study. Any presentations and publications will not identify you personally. We hope to use the information from this study to design a large trial of Morita therapy and potentially help us to treat future patients better.

What if something goes wrong or I have a complaint?

We do not expect any harm coming to you from being in this study. However, if you wished to complain, or had any concerns about any aspect of the way you have been approached or were treated during the course of this study, the normal National Health Service complaints mechanisms are available to you through the Patient Advice and Liaison Service (PALS) on 0800 0730741. Alternatively, if you are randomised to receive Morita therapy, you may prefer to raise the matter with the Mood Disorders Centre AccEPT Clinic. Written complaints should be sent to the AccEPT clinic complaints manager at: Washington Singer Laboratories, School of Psychology, University of Exeter, Perry Road, Exeter, EX4 4QG. If

you are eligible, agree to take part, randomised to Morita therapy and are unhappy with the care or treatment you receive, you can also raise the matter (in writing or by speaking) with your clinic therapist.

Will my taking part in this study be kept confidential?

All information collected about you during the course of the research will be kept strictly confidential. Any personal details, such as name and address, that we collect from you will be stored securely for five years and accessed only by the study team. Any information about you that is collected from the questionnaires or interviews will be stored indefinitely on the University of Exeter's open access repository (Open Research Exeter) in order to support other research in the future. These will have all personal details removed so that you cannot be recognised from them.

As your GP may be involved in your treatment, s/he will be informed of your progress as part of the research study. Should your condition worsen to a point where it is felt by either a researcher or a clinician that you may be a danger to yourself or others, your GP will be informed of this; with or without your permission. However, this is the only time we would ever break confidentially.

In the unlikely event that you become unable to consent to taking part during the study, we will withdraw you from the study. We will retain any data which you have already given us but will only use this confidentially and in line with the consent you have already given us.

Who is organising and funding the research?

The study researcher is funded by the University of Exeter Medical School who also sponsor this research. This is not a commercially funded industry study. This means that your GP surgery and the Exeter Depression and Anxiety Service who may have invited you to express your interest in the study, and the research team, will not receive any extra money for conducting this study.

Who has reviewed the study?

All research involving NHS patients is looked at by an independent group of people called a Research Ethics Committee to protect your safety, rights well-being and dignity. The study

has been reviewed and given a favourable opinion by the South West - Frenchay Research Ethics Committee.

Further Information – Next Steps

Please look at the 'Participant Flow Chart' on the next page which sets out the assessment and treatment process in a way which we hope you find helpful. During our arranged meeting you will have the chance to ask questions and we will ask you for more information to find out if you are eligible. If you are eligible and want to take part, we will ask you to sign a form to say so and then get you to fill out some questionnaires about yourself.

Contact for Further Information

If you need further information or have any questions, please contact:

Holly Sugg, Morita Trial Researcher
University of Exeter Medical School
Room 1.33, South Cloisters
St Luke's Campus
Heavitree Road
Exeter EX1 2LU

Email: h.v.s.sugg@exeter.ac.uk
Office telephone: 01392 727412

Thank you for reading this and for considering taking part in this study.

Morita Trial Participant Flow Chart

You expressed your interest in taking part in a trial of a treatment called Morita therapy

You have spoken to the study researcher who has arranged an assessment meeting with you to find out if you are eligible to take part in the study

At the assessment the study will be explained to you fully and you can ask any questions you may have. You will be asked some questions to see if you are eligible

If you are eligible and happy to proceed you will be asked to provide your consent to take part in the study

If after the assessment you are found not to be eligible to enter into the study, we are very sorry and thank you very much for your interest in the Morita Trial. You will return to the usual care of your GP

If you are enrolled into the trial your details will be entered onto a secure computer that picks at random whether you will receive Morita therapy or treatment as usual

You may be invited to an interview about why you chose to take part

Morita therapy involves between 8 and 12 one hour one-to-one sessions with a therapist on a weekly basis

Treatment as usual means you will not receive treatment in the study but may access treatment elsewhere as normal

There will be a follow-up appointment at 4 months

You will be invited to an interview after you finish Morita therapy

Thank you. You have completed your involvement in the Morita Trial and we thank you for your participation. You will return to the usual care of your GP and will be sent a summary of the results of the Morita studies if you would like to
