# Supplementary material for: Morita therapy for depression and anxiety (Morita Trial): study protocol for a pilot randomised controlled trial
Source: Trials. 2016 Mar 24;17:161. doi: 10.1186/s13063-016-1279-3 (PMC4806496; doi:10.1186/s13063-016-1279-3)
Supplement: Additional file 4: — Model Consent Form. (PDF 246 kb) [file 13063_2016_1279_MOESM4_ESM.pdf]

The Morita Trial (Morita Therapy for Depression and Anxiety: A Feasibility and Pilot Study)

CONSENT FORM

This consent form is in two parts. You do not have to sign both parts.

Part 1 on page 1: This is about your general participation in the study. Please initial the boxes and sign if you agree to take part.

Part 2 on Page 2 is optional – you can choose if you wish to take part or not: This is about whether you agree to being interviewed by a study researcher about your reasons for taking part in the trial and, if you receive Morita therapy, your views of the therapy. This also asks whether you agree to your Morita therapy sessions being audio-recorded, if you receive Morita therapy in the study.

PART 1: MAIN STUDY	Please initial box
1. I confirm that I have read and understand the information leaflet (dated 26/05/15 (version 2) for the above study. I have had the opportunity to consider the information, ask questions and have had these answered satisfactorily.	
2. I understand that my participation is voluntary and that I am free to withdraw at any time, without giving any reason, without my medical care or legal rights being affected.	
3. I agree to my GP being informed of my participation in this study and updated with information from this study relevant to my medical care.	
4. I understand that relevant sections of my medical notes and data collected during the study may be looked at by individuals from regulatory authorities or from the NHS Trust, where it is relevant to my taking part in the research. I give permission for these individuals to have access to my records.	
5. I understand that the data collected for this study which may identify me personally will be retained securely for up to five years, even if I decide to withdraw from the study, and that it will be accessed by the study researchers only.	
5. I understand that the data collected for this study will be anonymised (the information which may identify me personally will be removed) and will be stored indefinitely in Open Research Exeter in order to support other research in the future.	
6. I agree to take part in the above study.	
7. I would like to receive a summary of results of the Morita therapy studies via [email] or [post] (please delete as appropriate, and delete both if you do not wish to receive a summary)	

When you have initialled the boxes above, please write your name and sign and date below.

**Name of Participant** *(BLOCK CAPITALS)*	**Date**	**Signature**

TO BE COMPLETED BY THE RESEARCHER:

I have explained the study to the above patient and he/she has indicated his/her willingness to take part in the study"

**Name of Researcher** *(BLOCK CAPITALS)*	**Date**	**Signature**

I copy for patient, 1 copy for researcher, 1 copy for GP

The Morita Trial (Morita Therapy for Depression and Anxiety: A Feasibility and Pilot Study)

PART 2: INTERVIEWS AND AUDIO-RECORDING (OPTIONAL)

This section is optional.

You can choose if you wish to be interviewed and/or have your Morita therapy sessions audio-recorded or not.

Your decision will not affect your participation in the main part of the study.

This consent form is about whether you agree or not to being interviewed and your interviews being audio-recorded. It also asks you whether you agree to your Morita therapy sessions being audio-recorded or not if you receive Morita therapy through the study, and how you are happy for us to use these recordings. Please note that if you agree either of these, your data will be retained and shared in accordance with the consent form for the main study.

Please only initial the boxes that you wish to consent to. Thank you.

	**Please initial box**
1. I am willing for my Morita therapy sessions to be audio recorded for clinical purposes.	
2. I am willing for my therapy session recordings to be retained for research purposes.	
3. I am willing for my therapy session recordings to be retained for training purposes.	
4. I am willing to be interviewed.	
5. I am willing for the interviews to be audio recorded for research purposes only and I understand that the recordings will be deleted once transcribed.	
6. I am willing for publications and presentations to include direct quotations from me which will be made anonymous.	

When you have initialled the boxes above, please write your name and sign and date below.

Name of Participant *(BLOCK CAPITALS)* **Date** **Signature**

TO BE COMPLETED BY THE RESEARCHER:

I have explained the additional part of study to the above patient and he/she has indicated which parts apply.

Name of Researcher *(BLOCK CAPITALS)* **Date** **Signature**

I copy for patient, 1 copy for researcher, 1 copy for GP
